# Supplementary material for: Hybrid Models and Biological Model Reduction with PyDSTool
Source: PLoS Comput Biol. 2012 Aug 9;8(8):e1002628. doi: 10.1371/journal.pcbi.1002628 (PMC3415397; doi:10.1371/journal.pcbi.1002628)
Supplement: Text S4 — Complete source code for the PyDSTool package (version 0.88.120504). Includes API documentation and help files linking to web pages. This file is identical to the current public release on Sourceforge.net. (ZIP) [file pcbi.1002628.s004.zip › PyDSTool/html/PyDSTool.Toolbox.neuralcomp-pysrc.html]

xml version="1.0" encoding="ascii"?


PyDSTool.Toolbox.neuralcomp


| Home | Trees | Indices | Help | PyDSTool |
| --- | --- | --- | --- | --- |

Package PyDSTool ::
Package Toolbox ::
Module neuralcomp

[hide private]

[frames] | no frames]

# Source Code for Module PyDSTool.Toolbox.neuralcomp

```
   1  """
 
   2      An example set of basic compartmental model ModelSpec classes
 
   3      for use in computational neuroscience modeling.
 
   4  
 
   5      Basic classes provided:
 
   6      channel, compartment
 
   7  
 
   8      For multi-compartment models:
 
   9      soma, dendr_compartment, synapse, neuron, network, exc_synapse, inh_synapse
 
  10  
 
  11  Rob Clewley, September 2005.
 
  12  """ 
  13  from __future__ import division 
  14  
 
  15  from PyDSTool import * 
  16  from copy import copy 
  17  
 
  18  # which generators will specs made from these classes be compatible with
 
  19  compatGens = findGenSubClasses('ODEsystem') 
  20  
 


21 -class compatODEComponent(Component):


22      compatibleGens=compatGens 
  23      targetLangs=targetLangs  # (from common.py) -- all are compatible with ODEs

  24  
 


25 -class compatODELeafComponent(LeafComponent):


26      compatibleGens=compatGens 
  27      targetLangs=targetLangs  # (from common.py) -- all are compatible with ODEs

  28  
 
  29  # -----------------------------------------------------------------------------
 
  30  
 
  31  # basic classes -- not intended for direct use
 
  32  # indicates that only ODE-based Generators are compatible
 


33 -class channel(compatODELeafComponent):


34      pass

  35  
 


36 -class compartment(compatODEComponent):


37      pass

  38  
 
  39  compartment.compatibleSubcomponents=(channel,) 
  40  channel.compatibleContainers=(compartment,) 
  41  
 


42 -class channel_on(channel):


43      pass

  44  
 


45 -class channel_off(channel):


46      pass

  47  
 
  48  
 
  49  # use these for multi-compartment cell model:
 


50 -class soma(compartment):


51      pass

  52  
 


53 -class dendr_compartment(compartment):


54      pass

  55  
 


56 -class neurite_compartment(compartment):


57      pass

  58  
 


59 -class neuron(compatODEComponent):


60      pass

  61  
 
  62  
 
  63  # coupling
 


64 -class synapse(compatODELeafComponent):


65      pass

  66  
 


67 -class exc_synapse(synapse):


68      pass

  69  
 


70 -class inh_synapse(synapse):


71      pass

  72  
 
  73  # point-neuron network
 


74 -class pnnetwork(compatODEComponent):


75      pass

  76  
 
  77  # regular network
 


78 -class network(compatODEComponent):


79      pass

  80  
 
  81  soma.compatibleContainers=(neuron, pnnetwork) 
  82  neuron.compatibleSubcomponents=(soma, dendr_compartment, neurite_compartment,
 
  83                                  synapse) 
  84  neuron.compatibleContainers=(network,) 
  85  synapse.compatibleContainers=(neuron, pnnetwork) 
  86  pnnetwork.compatibleSubcomponents=(soma, synapse) 
  87  network.compatibleSubcomponents=(neuron,) 
  88  
 
  89  # helpful defaults exported for end-users
 
  90  global voltage, V 
  91  voltage = 'V' 
  92  V = Var(voltage) 
  93  
 
  94  
 
  95  # ----------------------------------------------------------------------------
 
  96  ## Object factory functions, and related utilities
 
  97  
 


98 -def disconnectSynapse(syn, mspec):


99      """Disconnect synapse object syn from ModelSpec object mspec in
 
 100      which it has been declared, and remove its target cell's synaptic
 
 101      channels.""" 
 102      targsfound = [] 
 103      for targname in syn.connxnTargets: 
 104          targsfound.extend(searchModelSpec(mspec, targname)) 
 105      if len(targsfound) == len(syn.connxnTargets): 
 106          if remain(targsfound, syn.connxnTargets) == []: 
 107              # all targets were found
 
 108              mspec.remove(targsfound) 
 109          else: 
 110              raise ValueError("synapse targets were not found in model spec"
 
 111                               "argument") 
 112      else: 
 113          raise ValueError("synapse targets were not found in model spec"
 
 114                           "argument") 
 115      syn.delConnxnTarget(targsfound) 
 116      mspec.remove(syn)

 117  
 
 118  
 


119 -def connectWithSynapse(synname, syntypestr, source_cell, dest_cell,
 
 120                         dest_compartment_name="",
 
 121                         threshfun=None, alpha=None, beta=None,
 
 122                         threshfun_d=None, alpha_d=None, beta_d=None,
 
 123                         adapt_typestr=None, vrev=None, g=None,
 
 124                         noauxs=True, subclass=channel):


125      """Make a chemical or electrical synapse between two neurons. For
 
 126      gap junctions this function returns None, otherwise will return the
 
 127      synapse gating variable object.
 
 128  
 
 129      Valid source_cell and dest_cell is a neuron or, for point neurons only,
 
 130        a soma is allowed.
 
 131      The optional argument dest_compartment_name is a declared name in
 
 132      dest_cell, or by default will be the soma.
 
 133  
 
 134      The standard assumed rate equation for a chemical synapse conductance is
 
 135  
 
 136         s' = (1-alpha) * s - beta * s
 
 137  
 
 138      Use the _d versions of threshfun, alpha, and beta, for an adapting chemical
 
 139      synapse type's adapting variable.
 
 140      """ 
 141      if isinstance(source_cell, compartment): 
 142          addedNeuron1Name = "" 
 143          soma1name = source_cell.name 
 144      elif isinstance(source_cell, neuron): 
 145          addedNeuron1Name = source_cell.name + '.' 
 146          soma1name = source_cell._componentTypeMap['soma'][0].name 
 147      else: 
 148          raise TypeError("Invalid cell type to connect with synapse") 
 149      if isinstance(dest_cell, compartment): 
 150          addedNeuron2Name = "" 
 151          if dest_compartment_name == "": 
 152              comp2name = dest_cell.name 
 153          else: 
 154              raise ValueError("Cannot specify destination compartment name "
 
 155                               "when dest_cell has type soma") 
 156      elif isinstance(dest_cell, neuron): 
 157          addedNeuron2Name = dest_cell.name + '.' 
 158          if dest_compartment_name == "": 
 159              comp2name = dest_cell._componentTypeMap['soma'][0].name 
 160          else: 
 161              comp_objlist = dest_cell._componentTypeMap['compartment'] 
 162              comp_names = [o.name for o in comp_objlist] 
 163              # check legitimacy of dest_compartment_name in dest_cell
 
 164              if dest_cell._componentTypeMap['soma'][0].name == dest_compartment_name: 
 165                  comp2name = dest_compartment_name 
 166              elif dest_compartment_name in comp_names: 
 167                  comp2name = dest_compartment_name 
 168              else: 
 169                  raise ValueError("Destination compartment name " \
 
 170                                   + dest_compartment_name \
 
 171                                   + "not found in destination cell") 
 172      else: 
 173          raise TypeError("Invalid cell type to connect with synapse") 
 174  
 
 175      if addedNeuron1Name != "": 
 176          # we were given a neuron for source_cell, so should use its name
 
 177          # to help distinguish multiple occurrences of svar name at time
 
 178          # of registration in a network component.
 
 179          svarname1 = source_cell.name+"_"+soma1name 
 180      else: 
 181          svarname1 = soma1name 
 182      if addedNeuron2Name != "": 
 183          # we were given a neuron for dest_cell, so should use its name
 
 184          # to help distinguish multiple occurrences of svar name at time
 
 185          # of registration in a network component.
 
 186          svarname2 = dest_cell.name+"_"+comp2name 
 187      else: 
 188          svarname2 = comp2name 
 189  
 
 190      if syntypestr == 'gap': 
 191          # make the first 1/2 of the channel going from source -> dest
 
 192          channel_s12 = makeSynapseChannel(synname+'_1', None,
 
 193                                           comp2name+'.'+voltage,
 
 194                                           syntypestr, vrev=soma1name+'.'+voltage,
 
 195                                           g=g, noauxs=noauxs,
 
 196                                           subclass=subclass,
 
 197                                           gamma1={voltage: (synname+'_1',soma1name+'.'+voltage),
 
 198                                                   synname+'_1': (voltage,)}) 
 199          channel_name = nameResolver(channel_s12, dest_cell) 
 200          # update with globalized name
 
 201          targetChannel = addedNeuron2Name+comp2name+'.'+channel_name 
 202          channel_s12.name = channel_name 
 203          # make the other 1/2 channel going in the other direction
 
 204          channel_s21 = makeSynapseChannel(synname+'_2', None,
 
 205                                           soma1name+'.'+voltage,
 
 206                                           syntypestr, vrev=comp2name+'.'+voltage,
 
 207                                           g=g, noauxs=noauxs,
 
 208                                           subclass=subclass,
 
 209                                           gamma1={voltage: (synname+'_2',comp2name+'.'+voltage),
 
 210                                                   synname+'_2': (voltage,)}) 
 211          channel_name = nameResolver(channel_s21, source_cell) 
 212          # update with globalized name
 
 213          targetChannel = addedNeuron1Name+soma1name+'.'+channel_name 
 214          channel_s21.name = channel_name 
 215      else: 
 216          # chemical synapses
 
 217          svar = Var('s_'+svarname1+'_'+svarname2) 
 218          svarname = nameResolver(svar, dest_cell) 
 219          channel_s12 = makeSynapseChannel(synname,
 
 220                                           addedNeuron1Name+synname+'.'+svarname,
 
 221                                           comp2name+'.'+voltage,
 
 222                                           syntypestr, vrev=vrev, g=g, noauxs=noauxs,
 
 223                                           subclass=subclass,
 
 224                                           gamma1=args(voltage=(synname,),
 
 225                                                       synname=(voltage,))) 
 226          channel_name = nameResolver(channel_s12, dest_cell) 
 227          # update with globalized name
 
 228          targetChannel = addedNeuron2Name+comp2name+'.'+channel_name 
 229          channel_s12.name = channel_name 
 230          # check that this source synaptic gating variable hasn't been made before,
 
 231          # otherwise re-use that variable?
 
 232          if threshfun_d == alpha_d == beta_d == None: 
 233              syn12 = makeSynapse(synname, svarname,
 
 234                              addedNeuron1Name+soma1name+'.'+voltage,
 
 235                              syntypestr, threshfun=threshfun, alpha=alpha,
 
 236                              beta=beta, targetchannel=targetChannel, noauxs=noauxs) 
 237          else: 
 238              syn12 = makeAdaptingSynapse(synname, svarname, 'd',
 
 239                              addedNeuron1Name+soma1name+'.'+voltage,
 
 240                              syntypestr, adapt_typestr,
 
 241                              threshfun=threshfun, alpha=alpha,  beta=beta,
 
 242                              threshfun_d=threshfun_d, alpha_d=alpha_d, beta_d=beta_d,
 
 243                              targetchannel=targetChannel, noauxs=noauxs) 
 244      if addedNeuron2Name == "": 
 245          # we were given a soma for dest_cell
 
 246          dest_cell.add(channel_s12) 
 247      else: 
 248          comp = dest_cell.components[comp2name] 
 249          dest_cell.remove(comp2name) 
 250          comp.add(channel_s12) 
 251          dest_cell.add(comp) 
 252      if syntypestr == 'gap': 
 253          source_cell.addConnxnTarget(synname+'_1') 
 254          source_cell.add(channel_s21) 
 255          dest_cell.addConnxnTarget(synname+'_2') 
 256          return None 
 257      else: 
 258          source_cell.addConnxnTarget(synname) 
 259          if addedNeuron1Name != "": 
 260              # we were given a neuron class for source_cell, so we have to
 
 261              # add the synaptic variable component to that cell
 
 262              source_cell.add(syn12) 
 263          return syn12

 264  
 
 265  
 
 266  # ----------------------------------------------------------------------------
 
 267  
 
 268  
 


269 -def makeSynapseChannel(name, gatevarname=None, voltage=voltage, typestr=None,
 
 270                         vrev=None, g=None, noauxs=True, subclass=channel,
 
 271                         gamma1=None, gamma2=None):


272      """Make a chemical or electrical (gap junction) synapse channel in a soma.
 
 273      To select these, the typestr argument must be one of 'exc', 'inh', or 'gap'.
 
 274      For a user-defined chemical synapse use a different string name for typestr.
 
 275      For gap junctions, use the pre-synaptic membrane potential's soma ModelSpec
 
 276      object or its full hierarchical name string for the argument vrev.
 
 277  
 
 278      Returns a channel-type object by default, or some user-defined subclass if desired.
 
 279      """ 
 280      channel_s = subclass(name) 
 281      if vrev is None: 
 282          if typestr == 'gap': 
 283              raise ValueError("Must provide vrev for gap junctions") 
 284          elif typestr == 'exc': 
 285              vrevpar = Par('0', 'vrev') 
 286          elif typestr == 'inh': 
 287              vrevpar = Par('-75', 'vrev') 
 288          elif isinstance(typestr, str): 
 289              vrevpar = Par('vrev') 
 290          else: 
 291              raise TypeError("Invalid type for synapse") 
 292      elif typestr == 'gap': 
 293          if isinstance(vrev, str): 
 294              vrevpar = Var(vrev) 
 295          else: 
 296              vrevpar = vrev 
 297      elif isinstance(vrev, str): 
 298          vrevpar = Par(vrev, 'vrev') 
 299      elif isinstance(vrev, float) or isinstance(vrev, int): 
 300          vrevpar = Par(repr(vrev), 'vrev') 
 301      else: 
 302          # allow for ModelSpec function of other variables
 
 303          vrevpar = vrev 
 304      v_pot = Var(voltage)   # this is to provide the local name only 
 305      if g is None: 
 306          gpar = Par('g') 
 307      elif isinstance(g, str): 
 308          gpar = Par(g, 'g') 
 309      else: 
 310          gpar = Par(repr(g), 'g') 
 311      if typestr == 'gap': 
 312          condQ = gpar 
 313          if gatevarname is not None: 
 314              raise ValueError("gatevarname must be None for gap junctions") 
 315      else: 
 316          gatevar = Var(gatevarname) 
 317          condQ = gpar*gatevar 
 318  
 
 319      if noauxs: 
 320          I = Var(condQ*(v_pot-vrevpar), 'I', specType='ExpFuncSpec') 
 321          if typestr == 'gap': 
 322              # don't include vrevpar, which is really an already existing
 
 323              # V variable in a different cell
 
 324              channel_s.add([gpar,I]) 
 325          else: 
 326              channel_s.add([gpar,vrevpar,I]) 
 327      else: 
 328          cond = Var(condQ, 'cond', [0,Inf], specType='ExpFuncSpec') 
 329          shunt = Var(cond*vrevpar, 'shunt', specType='ExpFuncSpec') 
 330          I = Var(cond*(v_pot-vrevpar), 'I', specType='ExpFuncSpec') 
 331          if typestr == 'gap': 
 332              arg_list = [] 
 333          else: 
 334              arg_list = [gatevarname] 
 335          if not isinstance(vrevpar, Par): 
 336              if parlist is not None: 
 337                  avoid_pars = [str(q) for q in parlist if isinstance(q, Par)] 
 338              else: 
 339                  avoid_pars = [] 
 340              arg_list.extend(remain(vrevpar.freeSymbols, avoid_pars)) 
 341          cond_fn = Fun(condQ(), arg_list, 'dssrt_fn_cond') 
 342          shunt_fn = Fun(condQ()*vrevpar, arg_list, 'dssrt_fn_shunt') 
 343          I_fn = Fun(condQ()*(v_pot-vrevpar), arg_list+[voltage],
 
 344                     'dssrt_fn_I') 
 345          channel_s.add([gpar,I,cond,shunt,cond_fn,shunt_fn,I_fn]) 
 346          if typestr != 'gap': 
 347              # don't include vrevpar, which is really an already existing
 
 348              # V variable in a different cell
 
 349              channel_s.add([vrevpar]) 
 350          if gamma1 is None: 
 351              gamma1 = {} 
 352          if gamma2 is None: 
 353              gamma2 = {} 
 354          channel_s.gamma1 = gamma1 
 355          channel_s.gamma2 = gamma2 
 356      return channel_s

 357  
 
 358  
 


359 -def makeExtInputCurrentChannel(name, noauxs=True, subclass=channel,
 
 360                                 gamma1=None, gamma2=None):


361      """External input signal used directly as a current. Supply the Input having
 
 362      coordinate name 'ext_input'.
 
 363  
 
 364      Returns a channel-type object by default, or some user-defined subclass if desired.
 
 365      """ 
 366      channel_Iext = subclass(name) 
 367      # Input doesn't need a name for the internal signal (confused ModelSpec as an
 
 368      # unresolved name!)
 
 369      inp = Input('', 'ext_input', [0,Inf], specType='ExpFuncSpec') 
 370      I_ext = Var(-inp, 'I', specType='ExpFuncSpec') 
 371      if noauxs: 
 372          channel_Iext.add([inp, I_ext]) 
 373      else: 
 374          arg_list = ['ext_input'] 
 375          I_cond = Var('0', 'cond', [0,Inf], specType='ExpFuncSpec')   # no conductance for this plain current 
 376          I_shunt = Var(inp, 'shunt', specType='ExpFuncSpec')    # technically not a shunt but acts like a V-independent shunt! 
 377          cond_fn = Fun(inp(), arg_list, 'dssrt_fn_cond') 
 378          shunt_fn = Fun(inp()*vrevpar, arg_list, 'dssrt_fn_shunt') 
 379          I_fn = Fun(inp()*(v_pot-vrevpar), arg_list+[voltage],
 
 380                     'dssrt_fn_I') 
 381          channel_Iext.add([inp, I_ext, I_cond, I_shunt,
 
 382                            cond_fn, shunt_fn, I_fn]) 
 383          if gamma1 is not None: 
 384              raise ValueError("gamma1 must be None") 
 385          channel_Iext.gamma1 = {} 
 386          if gamma2 is None: 
 387              gamma2 = {} 
 388          channel_Iext.gamma2 = gamma2 
 389      return channel_Iext

 390  
 
 391  
 


392 -def makeExtInputConductanceChannel(name, voltage=voltage,
 
 393                                     g=None, vrev=None,
 
 394                                     noauxs=True, subclass=channel,
 
 395                                     gamma1=None, gamma2=None):


396      """External input signal used as a conductance. Supply the Input having
 
 397      coordinate name 'ext_input'.
 
 398  
 
 399      Returns a channel-type object by default, or some user-defined subclass if desired.
 
 400      """ 
 401      if g is None: 
 402          gpar = Par('g') 
 403      elif isinstance(g, str): 
 404          gpar = Par(g, 'g') 
 405      else: 
 406          gpar = Par(repr(g), 'g') 
 407      if vrev is None: 
 408          vrevpar = Par('vrev') 
 409      elif isinstance(vrev, str): 
 410          vrevpar = Par(vrev, 'vrev') 
 411      elif isinstance(vrev, float) or isinstance(vrev, int): 
 412          vrevpar = Par(repr(vrev), 'vrev') 
 413      else: 
 414          # allow for ModelSpec function of other variables
 
 415          vrevpar = vrev 
 416      if hasattr(vrevpar, 'name'): 
 417          declare_list = [gpar, vrevpar] 
 418      else: 
 419          declare_list = [gpar] 
 420      channel_Iext = subclass(name) 
 421      v_pot = Var(voltage)   # this is to provide the local name only 
 422      # Input doesn't need a name for the internal signal (confused ModelSpec as an
 
 423      # unresolved name!)
 
 424      inp = Input('', 'ext_input', [0,Inf], specType='ExpFuncSpec') 
 425      I_ext = Var(gpar*inp*(v_pot-vrevpar), 'I', specType='ExpFuncSpec') 
 426      if noauxs: 
 427          channel_Iext.add([inp, I_ext, gpar, vrevpar]) 
 428      else: 
 429          I_cond = Var(gpar*inp, 'cond', [0,Inf], specType='ExpFuncSpec')   # no conductance for this plain current 
 430          I_shunt = Var(I_cond*vrevpar, 'shunt', specType='ExpFuncSpec')    # technically not a shunt but acts like a V-independent shunt! 
 431          arg_list = ['ext_input'] 
 432          if not isinstance(vrevpar, Par): 
 433              if parlist is not None: 
 434                  avoid_pars = [str(q) for q in parlist if isinstance(q, Par)] 
 435              else: 
 436                  avoid_pars = [] 
 437              arg_list.extend(remain(vrevpar.freeSymbols, avoid_pars)) 
 438          cond_fn = Fun(gpar*inp(), arg_list, 'dssrt_fn_cond') 
 439          shunt_fn = Fun(gpar*inp()*vrevpar, arg_list, 'dssrt_fn_shunt') 
 440          I_fn = Fun(gpar*inp()*(v_pot-vrevpar), arg_list+[voltage],
 
 441                     'dssrt_fn_I') 
 442          channel_Iext.add([inp, I_ext, I_cond, I_shunt, gpar, vrevpar,
 
 443                            cond_fn, shunt_fn, I_fn]) 
 444          if gamma1 is not None: 
 445              raise ValueError("gamma1 must be None") 
 446          channel_Iext.gamma1 = {} 
 447          if gamma2 is None: 
 448              gamma2 = {} 
 449          channel_Iext.gamma2 = gamma2 
 450      return channel_Iext

 451  
 
 452  
 


453 -def makeBiasChannel(name, I=None, noauxs=True, subclass=channel,
 
 454                      gamma1=None, gamma2=None):


455      """Constant bias / applied current "channel".
 
 456  
 
 457      Returns a channel-type object by default, or some user-defined subclass if desired.
 
 458      """ 
 459      channel_Ib = subclass(name) 
 460      if I is None: 
 461          Ibias = Par('Ibias') 
 462      elif isinstance(I, str): 
 463          Ibias = Par(I, 'Ibias') 
 464      else: 
 465          Ibias = Par(repr(I), 'Ibias') 
 466      Ib = Var(-Ibias, 'I', specType='ExpFuncSpec') 
 467      if noauxs: 
 468          channel_Ib.add([Ibias,Ib]) 
 469      else: 
 470          Ibias_cond = Var('0', 'cond', [0,Inf], specType='ExpFuncSpec')   # no conductance for this plain current 
 471          Ibias_shunt = Var(Ibias, 'shunt', specType='ExpFuncSpec')    # technically not a shunt but acts like a V-independent shunt! 
 472          cond_fn = Fun('0', [], 'dssrt_fn_cond') 
 473          shunt_fn = Fun(Ibias, [], 'dssrt_fn_shunt') 
 474          I_fn = Fun(Ibias, [], 'dssrt_fn_I') 
 475          channel_Ib.add([Ibias,Ibias_cond,Ibias_shunt,Ib, cond_fn, shunt_fn, I_fn]) 
 476          if gamma1 is not None: 
 477              raise ValueError("gamma1 must be None") 
 478          channel_Ib.gamma1 = {} 
 479          if gamma2 is None: 
 480              gamma2 = {} 
 481          channel_Ib.gamma2 = gamma2 
 482      return channel_Ib

 483  
 
 484  
 


485 -def makeChannel_halfact(name,voltage=voltage,s=None,isinstant=False,sinf=None,
 
 486                  taus=None,spow=1,s2=None,isinstant2=False,sinf2=None,taus2=None,
 
 487                  spow2=1,vrev=None,g=None,
 
 488                  parlist=None, noauxs=True, subclass=channel,
 
 489                  gamma1=None, gamma2=None):


490      """Make an ionic membrane channel using the steady state and rate function formalism.
 
 491  
 
 492      i.e., that the gating variable s has a differential equation in the form:
 
 493         s' = (sinf(v) - v)/taus(v)
 
 494      The channel may have up to two gating variables, each of which is given by an ODE.
 
 495  
 
 496      If either gating variable has its corresponding 'isinstant' argument set to True, then
 
 497      that variable is set to be instananeous (algebraic, not an ODE), i.e. s = sinf(v). The
 
 498      taus function will then be ignored.
 
 499  
 
 500      The resulting channel current will be of the form
 
 501          name.I = g * s^spow * s2^spow2 * (voltage - vrev)
 
 502  
 
 503      Provide any additional Par or Fun objects necessary for the complete definition of the
 
 504      channel kinetics in the optional parlist argument.
 
 505  
 
 506      Returns a channel-type object by default, or some user-defined subclass if desired.
 
 507      """ 
 508      if g is None: 
 509          gpar = Par('g') 
 510      elif isinstance(g, str): 
 511          gpar = Par(g, 'g') 
 512      else: 
 513          gpar = Par(repr(g), 'g') 
 514      if vrev is None: 
 515          vrevpar = Par('vrev') 
 516      elif isinstance(vrev, str): 
 517          vrevpar = Par(vrev, 'vrev') 
 518      elif isinstance(vrev, float) or isinstance(vrev, int): 
 519          vrevpar = Par(repr(vrev), 'vrev') 
 520      else: 
 521          # allow for ModelSpec function of other variables
 
 522          vrevpar = vrev 
 523      if hasattr(vrevpar, 'name'): 
 524          declare_list = [gpar, vrevpar] 
 525      else: 
 526          declare_list = [gpar] 
 527      thischannel = subclass(name) 
 528      v_pot = Var(voltage)   # this is to provide the local name only 
 529      # deal with gating variable, if present
 
 530      if s is None: 
 531          condQ = gpar 
 532          assert taus==sinf==None 
 533          assert spow==1, "power should be unused when gating variable is None" 
 534          # override
 
 535          spow=0 
 536      else: 
 537          if taus==sinf==None: 
 538              assert isinstant == False 
 539              gatevar = Par(s) 
 540          else: 
 541              if isinstance(sinf, QuantSpec): 
 542                  sinf_term = Var(sinf(), name=s+'inf') 
 543              elif isinstance(sinf, str): 
 544                  sinf_term = Var(sinf, name=s+'inf') 
 545              else: 
 546                  raise TypeError("sinf argument must be a string or a QuantSpec") 
 547              if isinstant: 
 548                  if noauxs: 
 549                      gatevar = Var(sinf_term(), name=s, domain=[0,1],
 
 550                                   specType='ExpFuncSpec') 
 551                  else: 
 552                      taus_term = Var('0', name='tau'+s) 
 553                      taus_fn = Fun( '0', [v_pot], 'dssrt_fn_tau'+s ) 
 554                      sinf_fn = Fun( sinf_term(), [v_pot],
 
 555                                  'dssrt_fn_'+s+'inf' ) 
 556                      gatevar = Var(sinf_term(), name=s, domain=[0,1],
 
 557                                   specType='ExpFuncSpec') 
 558              else: 
 559                  if isinstance(taus, QuantSpec): 
 560                      taus_term = Var(taus(), name='tau'+s) 
 561                  elif isinstance(taus, str): 
 562                      taus_term = Var(taus, name='tau'+s) 
 563                  else: 
 564                      raise TypeError("taus argument must be a string or a QuantSpec") 
 565                  # temporary declaration of symbol s (the argument string) as a Var
 
 566                  s_ = Var(s) 
 567                  if noauxs: 
 568                      gatevar = Var( (sinf_term() - s_) / taus_term(), name=s,
 
 569                                   domain=[0,1], specType='RHSfuncSpec') 
 570                  else: 
 571                      taus_fn = Fun( taus_term(), [v_pot], 'dssrt_fn_tau'+s ) 
 572                      sinf_fn = Fun( sinf_term(), [v_pot],
 
 573                                  'dssrt_fn_'+s+'inf' ) 
 574                      gatevar = Var( (sinf_term() - s_) / taus_term(), name=s,
 
 575                                   domain=[0,1], specType='RHSfuncSpec') 
 576              if not noauxs: 
 577                  declare_list.extend([taus_term,sinf_term,taus_fn,sinf_fn]) 
 578          declare_list.append(gatevar) 
 579          condQ = gpar*makePowerSpec(gatevar,spow) 
 580  
 
 581      # deal with second gating variable, if present
 
 582      if s2 is not None: 
 583          assert s is not None, "Must use first gating variable name first!" 
 584          assert spow2 != 0, "Do not use second gating variable with spow2 = 0!" 
 585          s2_ = Var(s2) 
 586          if isinstance(sinf2, QuantSpec): 
 587              sinf2_term = Var(sinf2(), name=s2+'inf', specType='ExpFuncSpec') 
 588          elif isinstance(sinf2, str): 
 589              sinf2_term = Var(sinf2, name=s2+'inf', specType='ExpFuncSpec') 
 590          else: 
 591              raise TypeError("sinf2 argument must be a string or a QuantSpec") 
 592          if isinstant2: 
 593              if noauxs: 
 594                  gatevar2 = Var(sinf2_term(), name=s2, domain=[0,1],
 
 595                                specType='ExpFuncSpec') 
 596              else: 
 597                  taus2_term = Var('0', name='tau'+s2) 
 598                  taus2_fn = Fun( '0', [v_pot], 'dssrt_fn_tau'+s2 ) 
 599                  sinf2_fn = Fun( sinf2_term(), [v_pot],
 
 600                                  'dssrt_fn_'+s2+'inf' ) 
 601                  gatevar2 = Var(sinf2_term(), name=s2, domain=[0,1],
 
 602                                specType='ExpFuncSpec') 
 603          else: 
 604              if isinstance(taus2, QuantSpec): 
 605                  taus2_term = Var(taus2(), name='tau'+s2, specType='ExpFuncSpec') 
 606              elif isinstance(taus2, str): 
 607                  taus2_term = Var(taus2, name='tau'+s2, specType='ExpFuncSpec') 
 608              else: 
 609                  raise TypeError("taus2 argument must be a string or a QuantSpec") 
 610              if noauxs: 
 611                  gatevar2 = Var( (sinf2_term() - s2_) / taus2_term(), name=s2,
 
 612                                domain=[0,1], specType='RHSfuncSpec') 
 613              else: 
 614                  taus2_fn = Fun( taus2_term(), [v_pot], 'dssrt_fn_tau'+s2 ) 
 615                  sinf2_fn = Fun( sinf2_term(), [v_pot],
 
 616                                  'dssrt_fn_'+s2+'inf' ) 
 617                  gatevar2 = Var( (sinf2_term() - s2_) / taus2_term(), name=s2,
 
 618                                domain=[0,1], specType='RHSfuncSpec') 
 619          declare_list.append(gatevar2) 
 620          if not noauxs: 
 621              declare_list.extend([taus2_term, sinf2_term, taus2_fn, sinf2_fn]) 
 622          condQ = condQ * makePowerSpec(gatevar2, spow2) 
 623  
 
 624      if noauxs: 
 625          I = Var(condQ*(v_pot-vrevpar), 'I', specType='ExpFuncSpec') 
 626          declare_list.append(I) 
 627      else: 
 628          cond = Var(condQ, 'cond', [0,Inf], specType='ExpFuncSpec') 
 629          shunt = Var(cond*vrevpar, 'shunt', specType='ExpFuncSpec') 
 630          I = Var(cond*(v_pot-vrevpar), 'I', specType='ExpFuncSpec') 
 631          if s is None: 
 632              arg_list = [] 
 633          else: 
 634              arg_list = [s] 
 635          if s2 is not None: 
 636              arg_list.append(s2) 
 637          if not isinstance(vrevpar, Par): 
 638              if parlist is not None: 
 639                  avoid_pars = [str(q) for q in parlist if isinstance(q, Par)] 
 640              else: 
 641                  avoid_pars = [] 
 642              arg_list.extend(remain(vrevpar.freeSymbols, avoid_pars)) 
 643          cond_fn = Fun(condQ(), arg_list, 'dssrt_fn_cond') 
 644          shunt_fn = Fun(cond()*vrevpar, arg_list, 'dssrt_fn_shunt') 
 645          I_fn = Fun(cond()*(v_pot-vrevpar), arg_list+[voltage],
 
 646                     'dssrt_fn_I') 
 647          declare_list.extend([I,cond,shunt, cond_fn, shunt_fn, I_fn]) 
 648          if gamma1 is None: 
 649              gamma1 = {} 
 650          if gamma2 is None: 
 651              gamma2 = {} 
 652          thischannel.gamma1 = gamma1 
 653          thischannel.gamma2 = gamma2 
 654      if parlist is not None: 
 655          declare_list.extend(parlist) 
 656      thischannel.add(declare_list) 
 657      return thischannel

 658  
 
 659  
 


660 -def makeChannel_rates(name,voltage=voltage,
 
 661                        s=None,isinstant=False,arate=None,brate=None,spow=1,
 
 662                        s2=None,isinstant2=False,arate2=None,brate2=None,spow2=1,
 
 663                        vrev=None,g=None,parlist=None,noauxs=True,subclass=channel,
 
 664                        gamma1=None, gamma2=None):


665      """Make an ionic membrane channel using the forward and backward rate formalism.
 
 666  
 
 667      i.e., that the gating variable s has a differential equation in the form:
 
 668         s' = sa(v) * (1-s) - sb(v) * s
 
 669      The channel may have up to two gating variables, each of which is given by an ODE.
 
 670  
 
 671      If either gating variable has its corresponding 'isinstant' argument set to True, then
 
 672      that variable is set to be instananeous (algebraic, not an ODE),
 
 673      i.e. s = sinf(v) = a(v) / (a(v) + b(v))
 
 674  
 
 675      The resulting channel current will be of the form
 
 676          name.I = g * s^spow * s2^spow2 * (voltage - vrev)
 
 677  
 
 678      Provide any additional Par or Fun objects necessary for the complete definition of the
 
 679      channel kinetics in the optional parlist argument.
 
 680  
 
 681      Returns a channel-type object by default, or some user-defined subclass if desired.
 
 682      """ 
 683      if g is None: 
 684          gpar = Par('g') 
 685      elif isinstance(g, str): 
 686          gpar = Par(g, 'g') 
 687      else: 
 688          gpar = Par(repr(g), 'g') 
 689      if vrev is None: 
 690          vrevpar = Par('vrev') 
 691      elif isinstance(vrev, str): 
 692          vrevpar = Par(vrev, 'vrev') 
 693      elif isinstance(vrev, float) or isinstance(vrev, int): 
 694          vrevpar = Par(repr(vrev), 'vrev') 
 695      else: 
 696          # allow for ModelSpec function of other variables
 
 697          vrevpar = vrev 
 698      if hasattr(vrevpar, 'name'): 
 699          declare_list = [gpar, vrevpar] 
 700      else: 
 701          declare_list = [gpar] 
 702      thischannel = subclass(name) 
 703      v_pot = Var(voltage)   # this is to provide the local name only 
 704      # deal with gating variable, if present
 
 705      if s is None: 
 706          condQ = gpar 
 707          assert arate==brate==None 
 708          assert spow==1, "power should be unused when gating variable is None" 
 709          # override
 
 710          spow=0 
 711      else: 
 712          if arate==brate==None: 
 713              assert isinstant == False 
 714              gatevar = Par(s) 
 715          else: 
 716              if isinstance(arate, QuantSpec): 
 717                  aterm = Var(arate(), name='a'+s) 
 718              elif isinstance(arate, str): 
 719                  aterm = Var(arate, name='a'+s) 
 720              else: 
 721                  raise TypeError("arate argument must be a string or QuantSpec") 
 722              if isinstance(brate, QuantSpec): 
 723                  bterm = Var(brate(), name='b'+s) 
 724              elif isinstance(brate, str): 
 725                  bterm = Var(brate, name='b'+s) 
 726              else: 
 727                  raise TypeError("brate argument must be a string or QuantSpec") 
 728              # temporary declaration of symbol s (the argument string) as a Var
 
 729              s_ = Var(s) 
 730              if isinstant: 
 731                  if noauxs: 
 732                      gatevar = Var( aterm()/(aterm()+bterm()), name=s,
 
 733                                   domain=[0,1], specType='ExpFuncSpec') 
 734                  else: 
 735                      taus_term = Var('0', name='tau'+s) 
 736                      sinf_term = Var( aterm/(aterm+bterm), name=s+'inf') 
 737                      taus_fn = Fun( '0', [voltage], 'dssrt_fn_tau'+s ) 
 738                      sinf_fn = Fun( aterm()/(aterm()+bterm()), [voltage],
 
 739                                  'dssrt_fn_'+s+'inf' ) 
 740                      gatevar = Var( aterm()/(aterm()+bterm()), name=s,
 
 741                                   domain=[0,1], specType='ExpFuncSpec') 
 742              else: 
 743                  if noauxs: 
 744                      gatevar = Var( aterm()*(1-s_)-bterm()*s_, name=s,
 
 745                                   domain=[0,1], specType='RHSfuncSpec') 
 746                  else: 
 747                      taus_term = Var( 1/(aterm+bterm), name='tau'+s) 
 748                      sinf_term = Var( aterm*taus_term, name=s+'inf') 
 749                      taus_fn = Fun( 1/(aterm()+bterm()), [voltage],
 
 750                                     'dssrt_fn_tau'+s ) 
 751                      sinf_fn = Fun( aterm()/(aterm()+bterm()), [voltage],
 
 752                                  'dssrt_fn_'+s+'inf' ) 
 753                      gatevar = Var( aterm()*(1-s_)-bterm()*s_, name=s,
 
 754                                   domain=[0,1], specType='RHSfuncSpec') 
 755              if not noauxs: 
 756                  declare_list.extend([taus_term,sinf_term,aterm,bterm,
 
 757                                       taus_fn, sinf_fn]) 
 758          declare_list.append(gatevar) 
 759          condQ = gpar*makePowerSpec(gatevar,spow) 
 760  
 
 761      # deal with second gating variable, if present
 
 762      if s2 is not None: 
 763          assert s is not None, "Must use first gating variable name first!" 
 764          assert spow2 != 0, "Do not use second gating variable with spow2 = 0!" 
 765          if isinstance(arate2, QuantSpec): 
 766              aterm2 = Var(arate2(), name='a'+s2) 
 767          elif isinstance(arate2, str): 
 768              aterm2 = Var(arate2, name='a'+s2) 
 769          else: 
 770              raise TypeError("arate2 argument must be a string or QuantSpec") 
 771          if isinstance(brate2, QuantSpec): 
 772              bterm2 = Var(brate2(), name='b'+s2) 
 773          elif isinstance(brate2, str): 
 774              bterm2 = Var(brate2, name='b'+s2) 
 775          else: 
 776              raise TypeError("brate2 argument must be a string or QuantSpec") 
 777          s2_ = Var(s2) 
 778          if isinstant2: 
 779              if noauxs: 
 780                  gatevar2 = Var( aterm2()/(aterm2()+bterm2()), name=s2,
 
 781                                domain=[0,1], specType='ExpFuncSpec') 
 782              else: 
 783                  taus2_term = Var( '0', name='tau'+s2) 
 784                  sinf2_term = Var( aterm2()/(aterm2()+bterm2()), name=s2+'inf') 
 785                  taus2_fn = Fun( '0', [voltage], 'dssrt_fn_tau'+s2 ) 
 786                  sinf2_fn = Fun( aterm2()/(aterm2()+bterm2()), [voltage],
 
 787                                  'dssrt_fn_'+s2+'inf' ) 
 788                  gatevar2 = Var( aterm2()/(aterm2()+bterm2()), name=s2,
 
 789                                domain=[0,1], specType='ExpFuncSpec') 
 790          else: 
 791              if noauxs: 
 792                  gatevar2 = Var(aterm2()*(1-s2_)-bterm2()*s2_, name=s2,
 
 793                                 domain=[0,1], specType='RHSfuncSpec') 
 794              else: 
 795                  taus2_term = Var( 1/(aterm2()+bterm2()), name='tau'+s2) 
 796                  sinf2_term = Var( aterm2()*taus2_term(), name=s2+'inf') 
 797                  taus2_fn = Fun( 1/(aterm2()+bterm2()), [voltage], 'dssrt_fn_tau'+s2 ) 
 798                  sinf2_fn = Fun( aterm2()/(aterm2()+bterm2()), [voltage],
 
 799                                  'dssrt_fn_'+s2+'inf' ) 
 800                  gatevar2 = Var(aterm2()*(1-s2_)-bterm2()*s2_, name=s2,
 
 801                                 domain=[0,1], specType='RHSfuncSpec') 
 802          condQ = condQ * makePowerSpec(gatevar2, spow2) 
 803          declare_list.append(gatevar2) 
 804          if not noauxs: 
 805              declare_list.extend([taus2_term, sinf2_term, aterm2, bterm2,
 
 806                                   taus2_fn, sinf2_fn]) 
 807  
 
 808      if noauxs: 
 809          I = Var(condQ*(v_pot-vrevpar), 'I', specType='ExpFuncSpec') 
 810          declare_list.append(I) 
 811      else: 
 812          cond = Var(condQ, 'cond', [0,Inf], specType='ExpFuncSpec') 
 813          shunt = Var(cond*vrevpar, 'shunt', specType='ExpFuncSpec') 
 814          I = Var(cond*(v_pot-vrevpar), 'I', specType='ExpFuncSpec') 
 815          if s is None: 
 816              arg_list = [] 
 817          else: 
 818              arg_list = [s] 
 819          if s2 is not None: 
 820              arg_list.append(s2) 
 821          if not isinstance(vrevpar, Par): 
 822              if parlist is not None: 
 823                  avoid_pars = [str(q) for q in parlist if isinstance(q, Par)] 
 824              else: 
 825                  avoid_pars = [] 
 826              arg_list.extend(remain(vrevpar.freeSymbols, avoid_pars)) 
 827          cond_fn = Fun(condQ(), arg_list, 'dssrt_fn_cond') 
 828          shunt_fn = Fun(cond()*vrevpar, arg_list, 'dssrt_fn_shunt') 
 829          I_fn = Fun(cond()*(v_pot-vrevpar), arg_list+[voltage], 'dssrt_fn_I') 
 830          declare_list.extend([I,cond,shunt, cond_fn, shunt_fn, I_fn]) 
 831          if gamma1 is None: 
 832              gamma1 = {} 
 833          if gamma2 is None: 
 834              gamma2 = {} 
 835          thischannel.gamma1 = gamma1 
 836          thischannel.gamma2 = gamma2 
 837      if parlist is not None: 
 838          declare_list.extend(parlist) 
 839      thischannel.add(declare_list) 
 840      return thischannel

 841  
 
 842  
 


843 -def makeSoma(name, voltage=voltage, channelList=None, C=None, noauxs=True,
 
 844               subclass=soma, channelclass=channel):


845      """Build a soma type of "compartment" from a list of channels and a membrane
 
 846      capacitance C, using the local voltage name.
 
 847  
 
 848      Specify a sub-class for the channel to target specific types to summate,
 
 849      in case the user wants to have runtime control over which are "active" in the
 
 850      dynamics (mainly related to the DSSRT toolbox).
 
 851  
 
 852      Returns a soma-type object by default, or some user-defined subclass if desired.
 
 853      """ 
 854      channel_cls_name = className(channelclass) 
 855      v = Var(QuantSpec(str(voltage), '-for(%s,I,+)/C'%channel_cls_name, 'RHSfuncSpec'),
 
 856              domain=[-150,100]) 
 857      if C is None: 
 858          C = Par('C') 
 859      elif isinstance(C, str): 
 860          gpar = Par(C, 'C') 
 861      else: 
 862          C = Par(repr(C), 'C') 
 863  
 
 864      asoma = soma.__new__(subclass) 
 865      asoma.__init__(name) 
 866      declare_list = copy(channelList) 
 867      if noauxs: 
 868          declare_list.extend([v,C]) 
 869      else: 
 870          tauv = Var(QuantSpec('tauv', '1/(C*for(%s,cond,+))'%channel_cls_name)) 
 871          vinf = Var(QuantSpec('vinf', 'for(%s,shunt,+)'%channel_cls_name + \
 
 872                                         '/for(%s,cond,+)'%channel_cls_name)) 
 873          # for each cond in channel, append signature of dssrt_fn_cond
 
 874          arg_list_c = [] 
 875          arg_list_s = [] 
 876          c_sum = [] 
 877          s_sum = [] 
 878          dssrt_inputs = {} 
 879          vstr = str(voltage) 
 880          for c in channelList: 
 881              def process_targ_name(inp): 
 882                  if inp == vstr: 
 883                      return inp 
 884                  else: 
 885                      return c.name+'.'+inp

 886              for targ, inputs in c.gamma1.items(): 
 887                  proc_targ = process_targ_name(targ) 
 888                  if proc_targ not in dssrt_inputs: 
 889                      dssrt_inputs[proc_targ] = args(gamma1=[], gamma2=[]) 
 890                  dssrt_inputs[proc_targ].gamma1.extend([process_targ_name(i) \
 
 891                                                                       for i in inputs]) 
 892              for targ, inputs in c.gamma2.items(): 
 893                  proc_targ = process_targ_name(targ) 
 894                  if proc_targ not in dssrt_inputs: 
 895                      dssrt_inputs[proc_targ] = args(gamma1=[], gamma2=[]) 
 896                  dssrt_inputs[proc_targ].gamma2.extend([process_targ_name(i) \
 
 897                                                                       for i in inputs]) 
 898              cond = c['dssrt_fn_cond'] 
 899              shunt = c['dssrt_fn_shunt'] 
 900              cond_sig = cond.signature 
 901              shunt_sig = shunt.signature 
 902              arg_list_c.extend(cond_sig) 
 903              arg_list_s.extend(shunt_sig) 
 904              c_sum.append( c.name + '.' + cond.name+'('+','.join(cond_sig)+')' ) 
 905              s_sum.append( c.name + '.' + shunt.name+'('+','.join(shunt_sig)+')' ) 
 906          arg_list1 = makeSeqUnique(arg_list_c) 
 907          arg_list1.sort() 
 908          arg_list2 = makeSeqUnique(arg_list_c+arg_list_s) 
 909          arg_list2.sort() 
 910          tauv_fn = Fun('1/(C*(%s))'%'+'.join(c_sum), arg_list1,
 
 911                        'dssrt_fn_tau'+vstr) 
 912          vinf_fn = Fun('(%s)/(%s)'%('+'.join(s_sum), '+'.join(c_sum)),
 
 913                        arg_list2, 'dssrt_fn_'+vstr+'inf') 
 914          declare_list.extend([v,C,vinf,tauv,tauv_fn,vinf_fn]) 
 915          asoma.dssrt_inputs = dssrt_inputs 
 916      asoma.add(declare_list) 
 917      return asoma 
 918  
 
 919  
 
 920  
 


921 -def makeDendrite(name, voltage=voltage, channelList=None, C=None, noauxs=True,
 
 922               subclass=dendr_compartment, channelclass=channel):


923      """Build a dendrite type of "compartment" from a list of channels and a membrane
 
 924      capacitance C, using the local voltage name.
 
 925  
 
 926      Specify a sub-class for the channel to target specific types to summate,
 
 927      in case the user wants to have runtime control over which are "active" in the
 
 928      dynamics (mainly related to the DSSRT toolbox).
 
 929  
 
 930      Returns a dendrite-type object by default, or some user-defined subclass if desired.
 
 931      """ 
 932      channel_cls_name = className(channelclass) 
 933      v = Var(QuantSpec(str(voltage), '-for(%s,I,+)/C'%channel_cls_name, 'RHSfuncSpec'),
 
 934              domain=[-150,100]) 
 935      if C is None: 
 936          C = Par('C') 
 937      elif isinstance(C, str): 
 938          gpar = Par(C, 'C') 
 939      else: 
 940          C = Par(repr(C), 'C') 
 941  
 
 942      acomp = dendr_compartment.__new__(subclass) 
 943      acomp.__init__(name) 
 944      declare_list = copy(channelList) 
 945      if noauxs: 
 946          declare_list.extend([v,C]) 
 947      else: 
 948          tauv = Var(QuantSpec('tauv', '1/(C*for(%s,cond,+))'%channel_cls_name)) 
 949          vinf = Var(QuantSpec('vinf', 'for(%s,shunt,+)'%channel_cls_name + \
 
 950                                         '/for(%s,cond,+)'%channel_cls_name)) 
 951          declare_list.extend([v,C,vinf,tauv]) 
 952      acomp.add(declare_list) 
 953      return acomp

 954  
 
 955  
 


956 -def makeNeurite(name, voltage=voltage, channelList=None, C=None, noauxs=True,
 
 957               subclass=dendr_compartment, channelclass=channel):


958      """Build a neurite type of "compartment" from a list of channels and a membrane
 
 959      capacitance C, using the local voltage name.
 
 960  
 
 961      Specify a sub-class for the channel to target specific types to summate,
 
 962      in case the user wants to have runtime control over which are "active" in the
 
 963      dynamics (mainly related to the DSSRT toolbox).
 
 964  
 
 965      Returns a neurite-type object by default, or some user-defined subclass if desired.
 
 966      """ 
 967      channel_cls_name = className(channelclass) 
 968      v = Var(QuantSpec(str(voltage), '-for(%s,I,+)/C'%channel_cls_name, 'RHSfuncSpec'),
 
 969              domain=[-150,100]) 
 970      if C is None: 
 971          C = Par('C') 
 972      elif isinstance(C, str): 
 973          gpar = Par(C, 'C') 
 974      else: 
 975          C = Par(repr(C), 'C') 
 976  
 
 977      acomp = neurite_compartment.__new__(subclass) 
 978      acomp.__init__(name) 
 979      declare_list = copy(channelList) 
 980      if noauxs: 
 981          declare_list.extend([v,C]) 
 982      else: 
 983          tauv = Var(QuantSpec('tauv', '1/(C*for(%s,cond,+))'%channel_cls_name)) 
 984          vinf = Var(QuantSpec('vinf', 'for(%s,shunt,+)'%channel_cls_name + \
 
 985                                         '/for(%s,cond,+)'%channel_cls_name)) 
 986          declare_list.extend([v,C,vinf,tauv]) 
 987      acomp.add(declare_list) 
 988      return acomp

 989  
 
 990  
 


991 -def makePointNeuron(name, voltage=voltage, channelList=None, synapseList=None,
 
 992                      C=None, noauxs=True):


993      """Factory function for single compartment neurons ("point neurons").
 
 994  
 
 995      Returns a neuron type object from a list of ion channels
 
 996      and synapses (if any are defined). The ion channels will internally be built into
 
 997      a soma compartment type on the fly.
 
 998      """ 
 999      asoma = makeSoma('soma', voltage, channelList, C=C, noauxs=noauxs) 
1000      n = neuron(name) 
1001      n.add(asoma) 
1002      if synapseList is not None: 
1003          n.add(synapseList) 
1004      return n

1005  
 
1006  
 


1007 -def makePointNeuronNetwork(name, componentList):


1008      """Factory function returning a pnnetwork type object from a list of compatible
 
1009      components (somas and synapses).
 
1010      """ 
1011      net = pnnetwork(name) 
1012      net.add(componentList) 
1013      return net

1014  
 
1015  
 


1016 -def makeNeuronNetwork(name, neuronList):


1017      """Factory function returning a network type object from a list of compatible
 
1018      components (neurons). The neurons can be single or multi-compartmental.
 
1019  
 
1020      Currently untested!
 
1021      """ 
1022      net = network(name) 
1023      net.add(neuronList) 
1024      return net

1025  
 
1026  
 


1027 -def makeSynapse(name, gatevar, precompartment, typestr, threshfun=None,
 
1028                  alpha=None, beta=None, targetchannel=None,
 
1029                  evalopt=True, noauxs=True):


1030      """Make a chemical synapse channel object.
 
1031      """ 
1032      if targetchannel is None: 
1033          raise TypeError("Must provide name of synaptic channel object in "
 
1034                          "target cell's compartment") 
1035      if typestr == 'exc': 
1036          if alpha is None: 
1037              alpha = 10. 
1038          if beta is None: 
1039              beta = 0.5 
1040          syn = exc_synapse(name) 
1041      elif typestr == 'inh': 
1042          if alpha is None: 
1043              alpha = 1. 
1044          if beta is None: 
1045              beta = 0.1 
1046          syn = inh_synapse(name) 
1047      elif typestr == "": 
1048          syn = synapse(name) 
1049      else: 
1050          raise ValueError("Invalid type of synapse specified") 
1051      s_ = Var(gatevar) 
1052      if alpha is None: 
1053          a = Par('alpha') 
1054      elif isinstance(alpha, str): 
1055          gpar = Par(alpha, 'alpha') 
1056      else: 
1057          a = Par(repr(alpha), 'alpha') 
1058      if beta is None: 
1059          b = Par('beta') 
1060      elif isinstance(beta, str): 
1061          gpar = Par(beta, 'beta') 
1062      else: 
1063          b = Par(repr(beta), 'beta') 
1064      if threshfun is None: 
1065          f = Fun('0.5+0.5*tanh(v/4.)', ['v'], 'thresh') 
1066      else: 
1067          assert isinstance(threshfun, tuple), \
 
1068                 "threshfun must be pair (vname, funbody)" 
1069          if isinstance(threshfun[1], QuantSpec): 
1070              funbody = threshfun[1].specStr 
1071          elif isinstance(threshfun[1], str): 
1072              funbody = threshfun[1] 
1073          else: 
1074              raise TypeError("threshold function must be a string or a "
 
1075                              "QuantSpec") 
1076          if threshfun[0] not in funbody: 
1077              print "Warning: voltage name %s does not appear in function body!"%threshfun[0] 
1078          f = Fun(funbody, [threshfun[0]], 'thresh') 
1079      assert len(f.signature) == 1, \
 
1080             'threshold function must be a function of a single argument (voltage)' 
1081      if evalopt: 
1082          alpha_ = a*f.eval(**{f.signature[0]: precompartment}) 
1083      else: 
1084          alpha_ = a*f(precompartment) 
1085          syn.add(f) 
1086      s = Var(alpha_*(1-s_)-b*s_, name=gatevar,
 
1087              specType='RHSfuncSpec', domain=[0,1]) 
1088      syn.add([s,a,b]) 
1089      syn.addConnxnTarget(targetchannel) 
1090      if not noauxs: 
1091          sinf = Var( alpha_/(alpha_+b), name=gatevar+'inf') 
1092          taus = Var( 1/(alpha_+b), name='tau'+gatevar) 
1093          sinf_fn = Fun( alpha_/(alpha_+b), [precompartment], 'dssrt_fn_'+gatevar+'inf') 
1094          taus_fn = Fun( 1/(alpha_+b), [precompartment], 'dssrt_fn_tau'+gatevar) 
1095          syn.add([sinf,taus,sinf_fn,taus_fn]) 
1096      return syn

1097  
 
1098  
 


1099 -def makeAdaptingSynapse(name, gatevar, adaptvar, precompartment, typestr,
 
1100                  adapt_typestr,
 
1101                  threshfun=None, alpha=None, beta=None,
 
1102                  threshfun_d=None, alpha_d=None, beta_d=None,
 
1103                  targetchannel=None, evalopt=True, noauxs=True):


1104      """Make an adapting chemical synapse channel object.
 
1105      """ 
1106      if targetchannel is None: 
1107          raise TypeError("Must provide name of synaptic channel object in "
 
1108                          "target cell's compartment") 
1109      if typestr == 'exc': 
1110          if alpha is None: 
1111              alpha = 10. 
1112          if beta is None: 
1113              beta = 0.5 
1114          syn = exc_synapse(name) 
1115      elif typestr == 'inh': 
1116          if alpha is None: 
1117              alpha = 1. 
1118          if beta is None: 
1119              beta = 0.1 
1120          syn = inh_synapse(name) 
1121      elif typestr == "": 
1122          syn = synapse(name) 
1123      else: 
1124          raise ValueError("Invalid type of synapse specified") 
1125      # synaptic variable
 
1126      s_ = Var(gatevar) 
1127      if alpha is None: 
1128          a = Par('alpha') 
1129      elif isinstance(alpha, str): 
1130          a = Par(alpha, 'alpha') 
1131      else: 
1132          a = Par(repr(alpha), 'alpha') 
1133      if beta is None: 
1134          b = Par('beta') 
1135      elif isinstance(beta, str): 
1136          b = Par(beta, 'beta') 
1137      else: 
1138          b = Par(repr(beta), 'beta') 
1139      if threshfun is None: 
1140          f = Fun('0.5+0.5*tanh(v/4.)', ['v'], 'thresh') 
1141      else: 
1142          assert isinstance(threshfun, tuple), \
 
1143                 "threshfun must be pair (vname, funbody)" 
1144          if isinstance(threshfun[1], QuantSpec): 
1145              funbody = threshfun[1].specStr 
1146          elif isinstance(threshfun[1], str): 
1147              funbody = threshfun[1] 
1148          else: 
1149              raise TypeError("threshold function must be a string or a "
 
1150                              "QuantSpec") 
1151          assert threshfun[0] in funbody, \
 
1152                 "voltage name %s does not appear in function body!"%threshfun[0] 
1153          f = Fun(funbody, [threshfun[0]], 'thresh') 
1154      assert len(f.signature) == 1, \
 
1155             'threshold function must be a function of a single argument (voltage)' 
1156      if evalopt: 
1157          alpha_ = a*f.eval(**{f.signature[0]: precompartment}) 
1158      else: 
1159          alpha_ = a*f(precompartment) 
1160          syn.add(f) 
1161      # adaptive variable
 
1162      if adapt_typestr == 'f': 
1163          at = '' 
1164      elif adapt_typestr == 'd': 
1165          at = '-' 
1166      else: 
1167          raise ValueError("Invalid type for adapting synapse: use 'f' or 'd'") 
1168      d_ = Var(adaptvar) 
1169      if alpha_d is None: 
1170          a_d = Par('alpha_d') 
1171      elif isinstance(alpha_d, str): 
1172          a_d = Par(alpha_d, 'alpha_d') 
1173      else: 
1174          a_d = Par(repr(alpha_d), 'alpha_d') 
1175      if beta_d is None: 
1176          b_d = Par('beta_d') 
1177      elif isinstance(beta_d, str): 
1178          b_d = Par(beta_d, 'beta_d') 
1179      else: 
1180          b_d = Par(repr(beta_d), 'beta_d') 
1181      if threshfun_d is None: 
1182          f_d = Fun('0.5+0.5*tanh(%sv/4.)'%atype, ['v'], 'thresh') 
1183      else: 
1184          assert isinstance(threshfun_d, tuple), \
 
1185                 "threshfun must be pair (vname, funbody)" 
1186          if isinstance(threshfun_d[1], QuantSpec): 
1187              funbody_d = threshfun_d[1].specStr 
1188          elif isinstance(threshfun_d[1], str): 
1189              funbody_d = threshfun_d[1] 
1190          else: 
1191              raise TypeError("threshold function must be a string or a "
 
1192                              "QuantSpec") 
1193          assert threshfun_d[0] in funbody_d, \
 
1194                 "voltage name %s does not appear in function body!"%threshfun[0] 
1195          f_d = Fun(funbody_d, [threshfun_d[0]], 'thresh') 
1196      assert len(f_d.signature) == 1, \
 
1197             'threshold function must be a function of a single argument (voltage)' 
1198      if evalopt: 
1199          alpha_d_ = a_d*f_d.eval(**{f_d.signature[0]: precompartment}) 
1200      else: 
1201          alpha_d_ = a_d*f_d(precompartment) 
1202          syn.add(f_d) 
1203      d = Var(alpha_d_*(1-d_)-b_d*d_, name=adaptvar,
 
1204              specType='RHSfuncSpec', domain=[0,1]) 
1205      s = Var(alpha_*(d_-s_)-b*s_, name=gatevar,
 
1206              specType='RHSfuncSpec', domain=[0,1]) 
1207      syn.add([s,a,b,d,a_d,b_d]) 
1208      syn.addConnxnTarget(targetchannel) 
1209      if not noauxs: 
1210          sinf = Var( alpha_*d_/(alpha_+b), name=gatevar+'inf') 
1211          taus = Var( 1/(alpha_+b), name='tau'+gatevar) 
1212          dinf = Var( alpha_d_/(alpha_d_+b_d), name=adaptvar+'inf') 
1213          taud = Var( 1/(alpha_d_+b_d), name='tau'+adaptvar) 
1214          sinf_fn = Fun( ) 
1215          taus_fn = Fun( ) 
1216          syn.add([sinf,taus,dinf,taud,sinf_fn,taus_fn]) 
1217      return syn

1218  
 
1219  # -----------------------------------------------------------------------------
 
1220  
 
1221  ## Helper functions
 
1222  
 


1223 -def makePowerSpec(v, p):


1224      if isinstance(p, int): 
1225          if p < 0: 
1226              raise ValueError("power should be > 0") 
1227          elif p==0: 
1228              return "1" 
1229          elif p > 4: 
1230              return Pow(v, p) 
1231          else: 
1232              res = v 
1233              for i in range(p-1): 
1234                  res = res * v 
1235              return res 
1236      else: 
1237          return Pow(v, p)

1238  
 
1239  
 
1240  # the following functions will be removed if the calling order for Quantities
 
1241  # is changed (as it ought to be some day).
 
1242  
 


1243 -def makePar(parname, val=None):


1244      if val is None: 
1245          return Par(parname) 
1246      elif isinstance(val, str): 
1247          gpar = Par(val, parname) 
1248      else: 
1249          return Par(repr(val), parname)

1250  
 
1251  
 


1252 -def makeFun(funname, sig, defn):


1253      return Fun(defn, sig, funname)

1254
```

  


| Home | Trees | Indices | Help | PyDSTool |
| --- | --- | --- | --- | --- |

|  |  |
| --- | --- |
| Generated by Epydoc 3.0.1 on Fri May 4 15:24:14 2012 | http://epydoc.sourceforge.net |
